# Intranasal Adenoviral Vector Vaccination Induces Durable Antibody and T Cell Responses in the Respiratory Tract

**DOI:** 10.3390/vaccines14090793

**Published:** 2026-09-09

**Authors:** Woochan Lee, Junghwa Lee, Sun Min Lee, Eui Ho Kim

**Affiliations:** 1 Department of Advanced Drug Discovery & Development, University of Science and Technology (UST), Daejeon 34113, Republic of Korea; 2 Viral Immunology Laboratory, Institut Pasteur Korea, Seongnam 13488, Republic of Korea

**Keywords:** respiratory pathogen, intranasal vaccination, adenoviral vector, mucosal immunity

## Abstract

**Background/Objectives**: Respiratory pathogens such as influenza virus and SARS-CoV-2 pose major threats to global public health. Current vaccines provide limited mucosal protection against respiratory infection, while vaccine-induced immunity can wane over time. Intranasal vaccination has emerged as a promising approach to induce mucosal immunity, however relatively low immunogenicity and limited durability remain major challenges. Various strategies have therefore been explored to improve intranasal vaccine efficacy, including mucosal adjuvants and alternative vaccine platforms. In this study, we evaluated the magnitude and durability of systemic and mucosal immune responses induced by an adenoviral vector vaccine. **Methods**: Six-week-old female C57BL/6 mice were immunized intranasally or intramuscularly with a single dose of an adenoviral vector vaccine and compared with mice receiving one or two doses of an intranasal CpG-adjuvanted protein vaccine. Humoral immune responses in serum and BALF, as well as cellular immune responses in the mLNs, lungs, and blood, were evaluated. We further assessed Ova-specific CD8 T cells with a lung-resident phenotype by class I MHC-peptide tetramer staining combined with intravascular labeling, allowing their direct ex vivo identification. **Results**: A single dose of intranasal adenoviral vector vaccination elicited potent serum IgG and BALF IgA responses, together with antigen-specific CD8 T cell responses in the blood, mLNs, and lungs. Notably, these systemic and respiratory mucosal immune responses were sustained for up to 4 months after vaccination, with lung-resident CD8 T cell responses detected at this time point. In contrast, intramuscular adenoviral vector vaccination predominantly induced systemic immunity. Intranasal CpG-adjuvanted protein vaccination induced weaker responses after a single dose, while booster vaccination enhanced systemic antibody and cellular responses but showed limited induction of mucosal immunity. **Conclusions**: A single intranasal dose of Ad5-Ova induced systemic and respiratory mucosal immune responses comparable to those observed after two-dose Ova+CpG vaccination, while maintaining these responses for up to 4 months. These findings suggest adenoviral vectors as a promising platform for inducing long-lasting respiratory mucosal immunity.

## 1. Introduction

Respiratory pathogens, including influenza virus and SARS-CoV-2, pose major threats to global public health, as demonstrated by the 1918 influenza pandemic and the Coronavirus disease of 2019 (COVID-19) pandemic [1,2]. The burden of respiratory infections is particularly substantial in vulnerable populations, including older individuals and those with compromised immune function, who are at increased risk of severe disease [3]. Therefore, effective strategies to prevent both infection and disease caused by respiratory pathogens remain an important public health threats.

Since Edward Jenner established the concept of vaccination, prophylactic vaccines have become one of the most successful approaches for preventing infectious diseases [4]. Vaccines against influenza and COVID-19 have substantially reduced morbidity and mortality and have provided effective protection against severe disease [5,6]. However, breakthrough infections continue to occur, partly because of waning vaccine-induced immunity and the continuous genetic evolution of respiratory pathogens [7]. Most of these vaccines are administered intramuscularly (IM) and primarily induce systemic immune responses that are effective in reducing disease severity. However, intramuscular vaccination generally induces limited mucosal immunity at the respiratory surface [8], and vaccine-induced immunity can decline over time [9]. Because the respiratory tract is the primary site of entry for many respiratory pathogens, vaccines that induce durable immunity at both systemic and mucosal sites may provide more effective protection against infection and transmission.

To solve these limitations, intranasal (IN) vaccination has emerged as a promising approach to induce mucosal immunity at the site of viral entry. Such responses include secretory immunoglobulin A (sIgA) in the respiratory tract and lung-resident memory lymphocytes [10,11]. However, the development of effective intranasal vaccines remains challenging because of their relatively weak and short-lived mucosal immune responses [12,13]. These limitations may be associated with the tolerogenic nature of mucosal tissues, rapid antigen clearance and degradation, and insufficient activation of local antigen-presenting cells [13,14]. Therefore, approaches that enhance both the magnitude and durability of mucosal immunity are needed.

To overcome these challenges, various approaches have been proposed to enhance the magnitude and durability of mucosal immune responses, including the use of mucosal adjuvants and vaccine platforms with favorable immunological properties [15,16]. Among mucosal adjuvants, CpG oligodeoxynucleotides (CpG ODNs) have been extensively investigated in preclinical studies. For example, intranasal CpG-adjuvanted vaccines have been shown to enhance systemic IgG and mucosal IgA responses compared with antigen-only vaccination [17], and CpG ODNs have been investigated as adjuvants for intranasal vaccines against influenza and SARS-CoV-2 [18,19]. Another approach is the use of vaccine platforms capable of efficiently delivering antigens and inducing robust immune responses. Adenoviral vector vaccines, including Ad26.COV2.S, ChAdOx1, Ad5-nCoV, and the Ad26/Ad5-based Sputnik V and Sputnik Light, have undergone clinical development as intramuscular SARS-CoV-2 vaccines. Adenoviral vectors have also been investigated for mucosal vaccination; notably, BBV154 (iNCOVACC), a replication-deficient chimpanzee adenovirus-vectored intranasal SARS-CoV-2 vaccine, was approved for use in India and has demonstrated the induction of systemic and mucosal immune responses. In preclinical studies, intranasal BBV154 induced neutralizing antibodies and T cell responses in the respiratory tract and provided protection against SARS-CoV-2 infection in non-human primates [20]. Recent studies have further demonstrated the potential of adenoviral vectors for inducing respiratory mucosal immunity, particularly when administered as mucosal boosts following systemic priming. In nonhuman primates, mucosal boosting with adenoviral vaccines enhanced airway IgA, IgG, and T cell responses and provided protection against SARS-CoV-2 infection [21,22]. In mice, a single intranasal dose of a rhesus adenovirus-vectored influenza vaccine induced mucosal IgA and T cell responses that were detectable for 7 months [23]. However, a direct characterization of the magnitude and persistence of coordinated systemic and respiratory mucosal immune responses following a single intranasal adenoviral vector vaccination remains limited. Here, we investigated whether a single intranasal dose of a recombinant adenovirus serotype 5-vector encoding ovalbumin (Ad5-Ova) could induce sustained systemic and respiratory mucosal antibody and CD8 T cell responses and compared these responses with those induced by single- and prime–boost intranasal Ova+CpG vaccination over a 4 month period.

## 2. Materials and Methods

### 2.1. Animal Experiments

All animal experiments were approved by the Institutional Animal Care and Use Committee of Institut Pasteur Korea (Protocol No. IPK-25001). Mice were housed in an Animal Biosafety Level 2 (ABSL-2) facility. Six-week-old female wild-type C57BL/6 mice were purchased from Orientbio. For intranasal or intramuscular immunization and the submandibular bleeding procedure, mice were anesthetized with isoflurane inhalation. Before tissue and BALF collection, mice were euthanized with CO_2_.

### 2.2. Vaccination

The following materials were used for vaccination: endotoxin-free ovalbumin (Ova, Worthington, OH, USA), CpG ODN 2395 (CpG, Invivogen, CA, USA), Dulbecco’s Phosphate-Buffered Saline (DPBS, Welgene, Republic of Korea). The Ad5-Ova vector used in this study is E1/E3-deleted and contains an Ova expression cassette inserted into the E1 deletion region under the control of a cytomegalovirus (CMV) promoter. The Ad5-Ova vector was originally developed by T. Griffith (University of Minnesota, MN, USA) and was obtained from the University of Iowa Viral Vector Core Facility.

The mice were divided into the following groups according to the vaccine formulation, route of administration, and number of doses: (1) Naïve; (2) Ova+CpG IN (x1), receiving Ova (10 μg/mouse) mixed with CpG (10 μg/mouse) in a total volume of 20 μL via the intranasal route as a single vaccination; (3) Ova+CpG IN (x2), receiving the same formulation and dose via the intranasal route for both prime and boost vaccinations; (4) Ad5-Ova IN, receiving 7 × 10^8^ IFU/mouse in a total volume of 20 μL via the intranasal route as a single vaccination; and (5) Ad5-Ova IM, receiving 7 × 10^8^ IFU/mouse in a total volume of 50 μL via the intramuscular route as a single vaccination. The boost vaccination for the Ova+CpG IN (x2) group was administered 3 weeks after the primary vaccination (Figure S1).

### 2.3. Serum Collection

For mouse serum collection, blood was collected via submandibular bleeding. The collected blood samples were allowed to clot at 4 °C. After clotting, the blood was centrifuged at 10,000 rpm for 10 min. The resulting supernatant was collected and stored at −80 °C until further analysis.

### 2.4. Peripheral Blood Mononuclear Cell (PBMC) Isolation

Blood was collected into 5 mL tubes via submandibular bleeding containing a mixture of 0.5 mL 4% sodium citrate solution and 1.5 mL harvest buffer (RPMI 1640 media (Welgene, Republic of Korea) supplemented with 1% fetal bovine serum (FBS, Gibco, NY, USA) and 1% 1 M HEPES (Welgene, Republic of Korea)). Histopaque-1077 (2 mL; Sigma-Aldrich, MO, USA), pre-warmed to 37 °C, was carefully underlaid beneath the collected blood layer. The blood samples were centrifuged at 400× *g* for 20 min at room temperature with the brake off. Following centrifugation, the PBMC interface layer was transferred to a new tube containing 2 mL of harvest buffer. To wash the collected PBMCs, the suspension was centrifuged at 300× *g* for 10 min, and the resulting cell pellet was prepared for further analysis.

### 2.5. Intravascular Anti-CD90.2 Staining for Detection of Lung-Resident Lymphocytes

Before euthanasia and sample collection, a BD OptiBuild™ Rat Anti-mouse CD90.2 antibody (BD Biosciences, NJ, USA) was intravenously injected to distinguish vascular-circulating lymphocytes from lung tissue-resident lymphocytes. A total of 3 μg of anti-CD90.2 antibody diluted in 200 μL of DPBS was injected into the tail vein. After intravenous injection, mice were maintained for 3–5 min to allow labeling of blood-circulating lymphocytes, followed by euthanasia and lung tissue collection. Intravascular CD90.2^+^ lymphocytes were distinguished from tissue-resident CD90.2^−^ lymphocytes based on intravascular antibody labeling. Ova-specific CD8 T cells with a lung-resident memory phenotype were identified as CD44^+^ Ova-specific CD8 T cells that were CD90.2^−^ and CD69^+^.

### 2.6. BALF Collection

To collect BALF, an IV catheter (20 G, BD Biosciences, NJ, USA) was inserted into the trachea. Cold DPBS (1 mL) was injected into the trachea, and the lavage fluid was aspirated twice through the catheter. Approximately 0.8 mL of BALF was recovered and collected in a 1.5 mL tube. The collected BALF samples were centrifuged at 300× *g* for 5 min at 4 °C, and the supernatants were transferred to new 1.5 mL tubes and stored at −80 °C until further analysis.

### 2.7. Tissue Collection & Single-Cell Preparation

Mediastinal lymph nodes (mLNs), spleens, and lungs were collected from mice. The mLNs and spleens were collected into harvest buffer consisting of RPMI supplemented with 1% FCS and 10 mM HEPES and mashed through a 70 μm cell strainer. Lungs were harvested into GentleMACS C Tubes (Miltenyi Biotec, Germany) containing harvest buffer and collagenase type IV (~400 U/mL per tissue; Worthington). Lung dissociation was performed using a GentleMACS Octo Dissociator (Miltenyi Biotec, Germany) by incubating the tissue with collagenase at 37 °C for approximately 30 min under sequential mechanical disruption steps (mixing for 36 s at ~165 rpm, followed by active blending intervals at 2844 rpm). Collagenase digestion was terminated by adding 20 μL of 0.5 M EDTA. The dissociated lung tissues were filtered through a 40 μm cell strainer. Red blood cell (RBC) lysis was performed for the lung and spleen single-cell suspensions using RBC lysis buffer for 1 min at room temperature, with gentle agitation every 30 s.

### 2.8. Immunoglobulin ELISA

Briefly, 96-well high-binding EIA/RIA plates (Corning, NY, USA) were coated with 50 μL/well of 2 μg/mL OVA protein in DPBS overnight. The coated plates were washed twice with 200 μL/well of PBS containing 0.05% Tween 20 (PBS-T, Takara, Japan) and blocked with blocking buffer (1% blotting-grade blocker in PBS-T, Bio-Rad, CA, USA) for 2 h at 37 °C. Serum and BALF samples were serially diluted 4-fold in blocking buffer, added to the plates, and incubated at room temperature for 2 h. The plates were washed three times with PBS-T, and HRP-conjugated goat anti-mouse antibodies (anti-mouse IgG, IgG1, IgG2b, and IgA, SouthernBiotech, AL, USA) in blocking buffer were added, followed by incubation for 1.5 h at room temperature. After washing three times with PBS-T, the wells were developed by adding TMB substrate solution (OptEIA reagent set, BD Biosciences, San Jose, CA, USA). Finally, the reaction was stopped by the addition of stop solution (1 N HCl). Optical density at 450 nm was measured using a microplate reader (Victor 3, PerkinElmer, CT, USA). Antibody titers were determined by endpoint analysis. Serially diluted positive control samples were used to generate a reference titration curve, and biological samples were compared with this curve to assign relative endpoint antibody titers expressed as arbitrary units (A.U.) [24].

### 2.9. Cell Counting

After the single-cell preparation, 10 μL of sample was mixed with 10 μL of 0.4% trypan blue solution (Gibco, NY, USA), and 10 μL of the mixture was loaded into the TC10/TC20 counting slide double-chamber (Bio-rad). The cell count was measured using the TC20 automated cell counter (Bio-rad, CA, USA).

### 2.10. Ex Vivo T Cell Stimulation

Cells from the lymph nodes and lungs underwent ex vivo stimulation with Ova major histocompatibility complex (MHC) class I peptide (Ova_257–264_, SIINFEKL, Invivogen, CA, USA) and MHC class II peptide (Ova_323–339_, ISQAVHAAHAEINEAGR, Invivogen, CA, USA) with protein transport inhibitors GolgiStop and GolgiPlug (BD Biosciences, San Jose, CA, USA) in a complete RPMI media (RPMI 1640 media with 10% FBS, 1% Penicillin-Streptomycin (Welgene, Republic of Korea), 1% L-glutamine (Welgene, Republic of Korea), and 1% Sodium pyruvate (Welgene, Republic of Korea) in 96 round-bottomed well cell culture plates. Each well was loaded with 1 × 10^6^ cells. The plate was incubated at 37 °C for 5 h. After incubation, the stimulated cells were subjected to cell-surface and intracellular staining procedures.

### 2.11. Flow Cytometry

Cells from different tissues were transferred into 96-well round-bottom plates (1 × 10^6^ cells/well). Cell viability was assessed using the Zombie Aqua Fixable Viability Kit (BioLegend, CA, USA) before surface staining. Antibodies were diluted in FACS buffer (1% FBS in DPBS), and mouse Fc block (BD Biosciences, NJ, USA) was included to prevent nonspecific Fc receptor-mediated antibody binding. Surface staining antibodies included CD4 (RM4-5, BioLegend, CA, USA), PD-1 (J43, BD Biosciences, NJ, USA), CD62L (MEL-14, BioLegend, CA, USA), CD8a (53-6.7, BioLegend, CA, USA), CD44 (IM7, BioLegend, CA, USA), CD127 (HIL-7R-M21, BD Biosciences, NJ, USA), CD69 (H1.2F3, BioLegend), CXCR5 (L138D7, BioLegend), TCR β chain (H57-597, BioLegend), CD95 (Jo2, BD Biosciences, NJ, USA), CD19 (6D5, BioLegend), and GL7 (GL7, BioLegend, CA, USA). Ova-specific CD8 T cells were detected using an H-2K^b^ Ova 257–264 peptide tetramer obtained from the NIH Tetramer Core Facility (Emory University, GA, USA). Antibodies and tetramer were added at 100 μL/well and incubated at 4 °C for 1 h protected from light. Cells were then washed with FACS buffer and fixed with 4% paraformaldehyde in PBS (Biosolution, Republic of Korea).

For ex vivo T cell stimulation, cells were stained for surface markers after viability staining and Fc blocking using CD4 (RM4-5), CD8a (53-6.7), CD44 (IM7), and TCR β chain (H57-597). Following surface staining and washing, cells were fixed and permeabilized using the Cytofix/Cytoperm Kit (BD Biosciences, NJ, USA). Intracellular staining was performed with antibodies against IL-2 (JES6-5H4, BioLegend, CA, USA), TNF (Mab11, BD Biosciences, NJ, USA), and IFN-γ (XMG1.2, BioLegend, CA, USA) diluted in Perm/Wash buffer (BD Biosciences, NJ, USA). The intracellular staining cocktail was added at 100 μL/well and incubated at 4 °C for 1 h protected from light. Cells were washed with Perm/Wash buffer followed by FACS buffer.

Stained cells were analyzed using a CytoFLEX LX flow cytometer (Beckman Coulter, CA, USA), and data were analyzed using FlowJo software version 10 (BD Biosciences, NJ, USA).

### 2.12. Statistical Analysis

Statistical analyses were performed using Prism (v9.0) (GraphPad Software, CA, USA). Normality was assessed using the Shapiro–Wilk normality test (*p* > 0.05 was considered indicative of a normal distribution). Homogeneity of variance was examined using the Brown–Forsythe test. To assess the statistical significance of antibody levels and cellular immune responses between vaccinated groups, one-way ANOVA, Welch’s ANOVA, or the Kruskal–Wallis test was used, depending on the data normality and homogeneity of variance. For multiple comparison tests, Tukey’s (one-way ANOVA), Dunnett’s T3 (Welch’s ANOVA), or Dunn’s (Kruskal–Wallis test) multiple comparisons test was applied. A *p*-value < 0.05 was considered statistically significant. Longitudinal ELISA and blood OVA-specific CD8 T cell dynamics were analyzed using a mixed-effects model followed by Tukey’s multiple comparisons test.

## 3. Results

### 3.1. Short-Term Immunogenicity of Different Vaccination Strategies

To evaluate the short-term humoral and cellular immune responses induced by different vaccine platforms and routes of administration, 6-week-old female C57BL/6 mice were immunized with the model antigen Ova (Figure S1A). Mice received Ova protein with CpG adjuvant either once (Ova+CpG IN (x1)) or twice (Ova+CpG IN (x2)) via the IN route, or a single dose of Ad5-Ova administered via the IN (Ad5-Ova IN) or IM (Ad5-Ova IM) route. Because adenoviral vector vaccines can induce durable immune responses following a single dose, whereas protein-based vaccines commonly require prime–boost immunization, we evaluated both single- and two-dose CpG-adjuvanted Ova vaccination as comparators for the single-dose Ad5-Ova regimen. Vaccine-induced immune responses were assessed at 1, 2, 3, and 4 weeks after the primary immunization. In the Ova+CpG IN (x2) group, the 4-week time point corresponded to one week after the booster immunization.

#### 3.1.1. Antigen-Specific Antibody Responses in Serum and BALF

**Figure 1:**
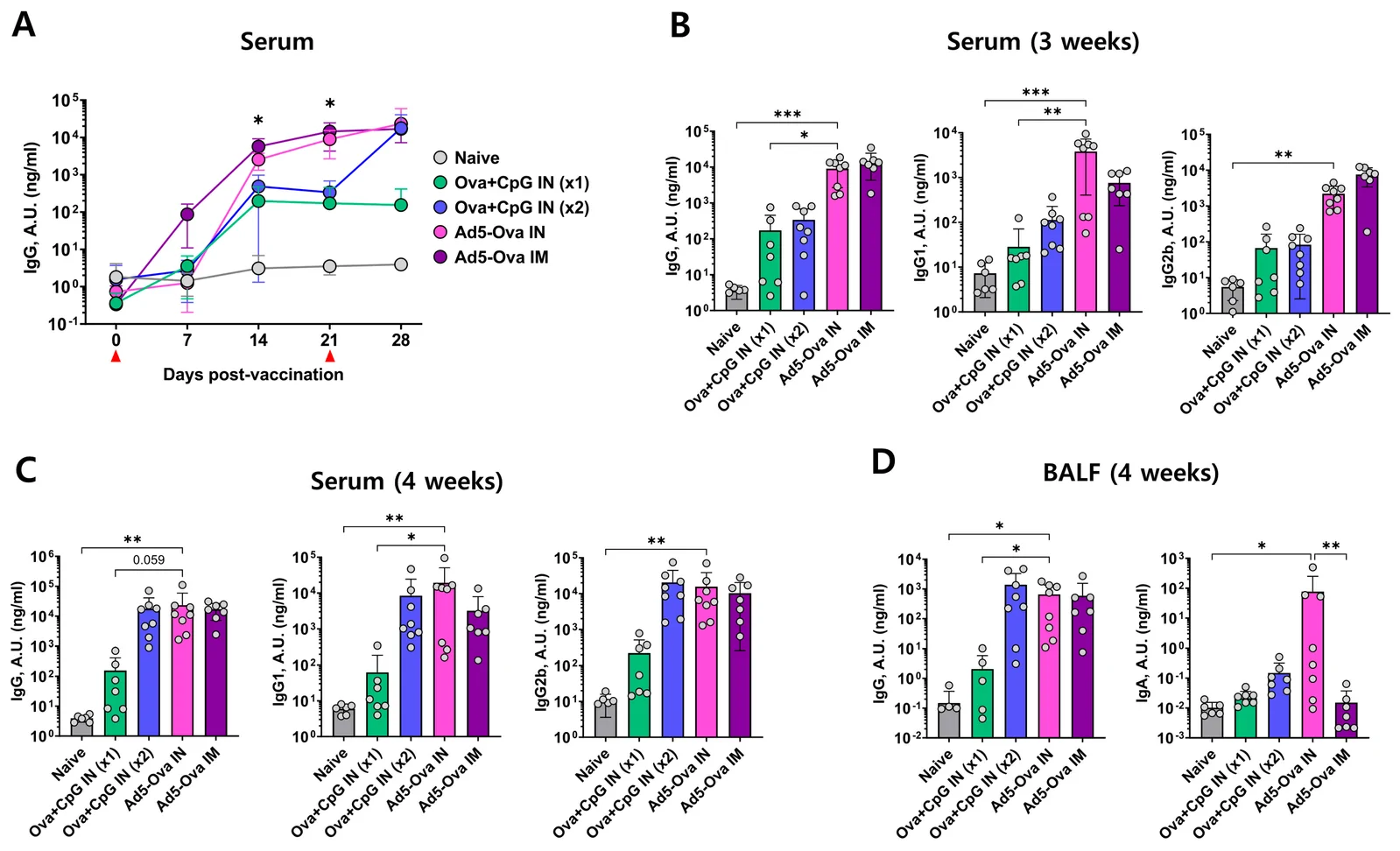
Antigen-specific antibody responses up to 4 weeks post-vaccination following intranasal or intramuscular vaccination with CpG-adjuvanted Ova protein or Ad5-Ova. (**A**) Kinetics of Ova-specific IgG responses in serum after vaccination. Red triangles indicate the day of vaccination. For statistical analysis, a mixed-effects model was used with Tukey’s multiple comparisons test. * *p* < 0.05, Ad5-Ova IN group vs. Ova+CpG IN (x2) group. (**B**) Ova-specific IgG, IgG1, and IgG2b levels in serum at 3 weeks post-primary vaccination. (**C**,**D**) Ova-specific IgG subset levels in serum (**C**) or IgG and IgA levels in BALF (**D**) at 4 weeks post-primary vaccination for the Ova+CpG IN (x1), Ad5-Ova IN, and Ad5-Ova IM groups, and 1 week post-boost vaccination for the Ova+CpG IN (x2) group. (**B**–**D**) For statistical analysis, the Kruskal–Wallis test was used with Dunn’s multiple comparisons test. Data are pooled from 2 independent experiments for Figure 1A–D. * *p* < 0.05; ** *p* < 0.01; *** *p* < 0.001. Data are presented as the mean ± SD.

To measure antigen-specific antibody responses, serum samples were collected before vaccination and at each post-vaccination time point. All immunized groups except the Ad5-Ova IM group showed induction of Ova-specific IgG responses from 2 weeks post-vaccination, whereas the Ad5-Ova IM group showed an earlier IgG response, detectable at 1 week post-vaccination Ad5-Ova IM (Figure 1A,B). Both Ad5-Ova IN and IM groups elicited the highest IgG levels, whereas IgG responses were weak after a single dose of Ova+CpG IN but increased to comparable levels after the second dose (Figure 1A,C).

A similar pattern was observed for Ova-specific IgG1 and IgG2b responses. The Ad5-Ova IN group showed robust responses after a single dose, whereas the Ova+CpG IN groups showed weaker responses that increased to comparable levels that were not significantly different from those in the Ad5-Ova IN group following the booster vaccination (Figure 1B,C).

To assess mucosal antibody responses in the respiratory tract, Ova-specific IgG and IgA levels in BALF were measured at 4 weeks after primary vaccination. BALF IgG was detected in the Ova+CpG IN (×2), Ad5-Ova IN, and Ad5-Ova IM groups (Figure 1D). In contrast, robust Ova-specific IgA responses were observed only in the Ad5-Ova IN group, whereas the other groups showed minimal or undetectable IgA levels (Figure 1D).

Together, these results demonstrate that a single intranasal dose of Ad5-Ova elicited robust systemic IgG and mucosal IgA responses. In contrast, the CpG-adjuvanted protein vaccine induced weaker systemic antibody responses after a single dose, which increased to levels comparable to Ad5-Ova following the booster dose, whereas mucosal IgA responses remained minimal. Intramuscular Ad5-Ova vaccination also failed to induce detectable mucosal IgA responses.

#### 3.1.2. Cellular Immune Responses in mLNs and Lungs

Next, mLNs and lungs were harvested to evaluate short-term cellular immune responses following vaccination. At 2 weeks after primary vaccination, Ad5-Ova IN vaccination induced enhanced activation of CD4 and CD8 T cells and Ova-specific CD8 T cell responses in the mLNs, the draining lymphoid tissues following IN immunization, whereas primary IN vaccination with CpG-adjuvanted Ova induced only marginal T cell responses (Figure S2A–C). Notably, Ad5-Ova IN vaccination significantly increased follicular helper T (Tfh) cell and germinal center (GC) B-cell responses in the mLNs, consistent with the robust mucosal IgA response observed following Ad5-Ova IN vaccination (Figure S2D,E).

In the lungs, a major respiratory effector site, Ad5-Ova IN vaccination induced more pronounced increases in activated T cells and Ova-specific CD8 T cells at 2 weeks post-vaccination compared with the other vaccination groups (Figure S3A–C). Intravascular staining with anti-CD90.2 antibody further demonstrated efficient establishment of lung-residency of Ova-specific CD8 T cells following Ad5-Ova IN vaccination compared with Ova+CpG IN and Ad5-Ova IM vaccination (Figure S3D).

**Figure 2:**
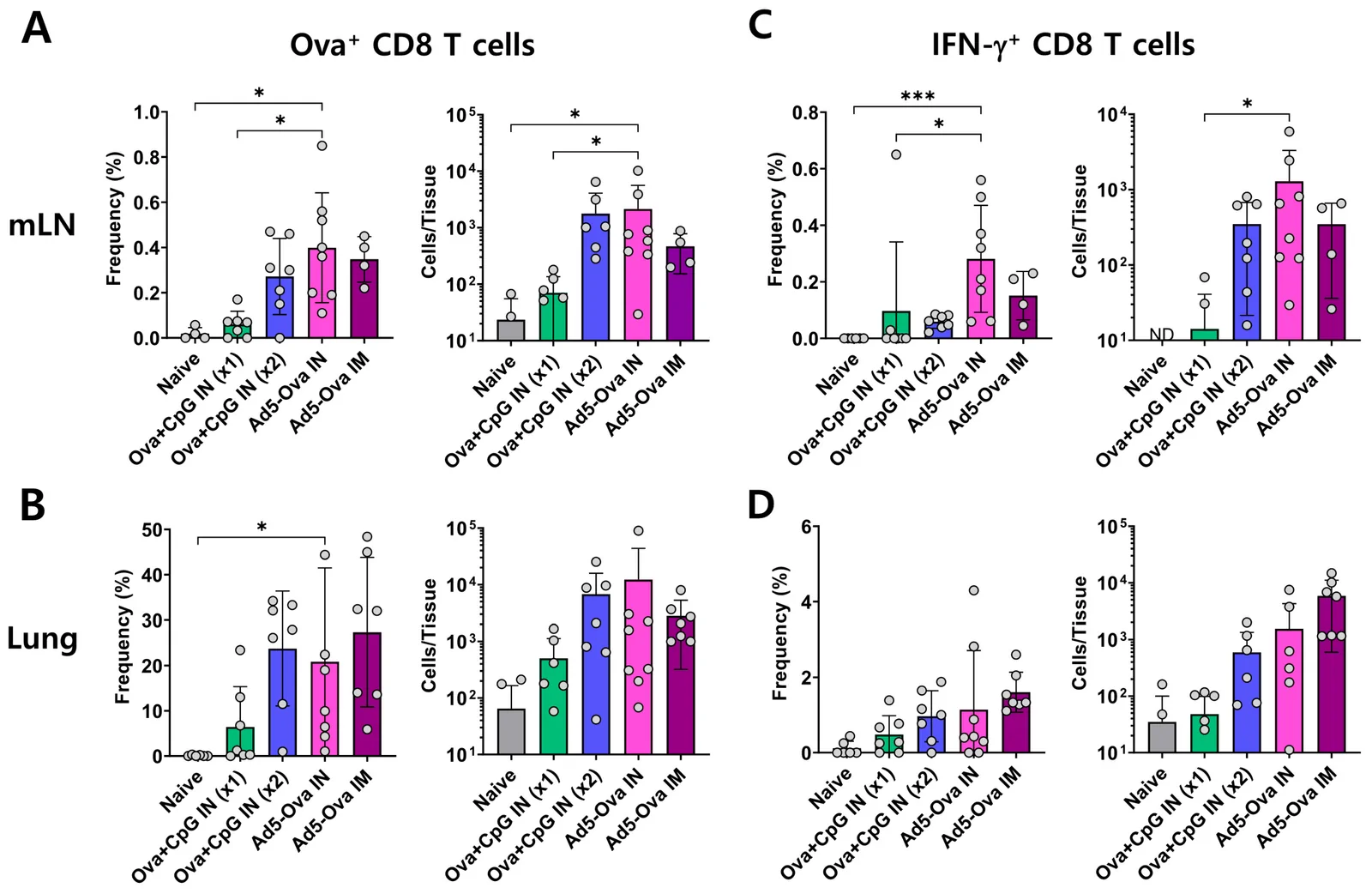
Ova-specific and IFN-γ-secreting CD8 T cell responses in the mLNs and lungs at 4 weeks post-primary vaccination. (**A**,**B**) Frequencies and numbers of Ova-specific (H2K^b^-Ova peptide tetramer^+^) CD8 T cells in the mLNs (**A**) and lungs (**B**). (**C**,**D**) Frequencies and numbers of IFN-γ-secreting CD8 T cells after ex vivo antigenic stimulation in the mLNs (**C**) and lungs (**D**). For statistical analysis, Welch’s ANOVA or the Kruskal–Wallis test was used, depending on normality and equality of variances. For multiple comparisons, Dunnett’s T3 test (Welch’s ANOVA) or Dunn’s test (Kruskal–Wallis test) was used. * *p* < 0.05; *** *p* < 0.001. Data are pooled from 2 independent experiments for Figure 2A–D. Data are presented as the mean ± SD.

At 4 weeks after primary vaccination (1 week after the booster vaccination in the Ova+CpG IN (×2) group), Ova-specific CD8 T cells, measured by class I MHC-peptide tetramer staining, were detected at high levels in the mLNs and lungs of all immunized groups except the Ova+CpG IN (×1) group (Figure 2A,B). To assess the functionality of antigen-specific CD8 T cells, cells isolated from the mLNs and lungs were ex vivo stimulated with SIINFEKL, an Ova-derived class I MHC peptide. The Ova+CpG IN (×2), Ad5-Ova IN, and Ad5-Ova IM groups tended to show increased numbers of IFN-γ-producing CD8 T cells in both tissues, consistent with the tetramer-staining results (Figure 2C,D).

Together, these results demonstrate that intranasal Ad5-Ova vaccination induced robust and functional antigen-specific CD8 T cell responses in the mLNs and lungs, along with Tfh and GC B-cell responses in the mLNs. These responses remained sustained at 4 weeks, whereas CpG-adjuvanted Ova vaccination required a booster dose to achieve comparable cellular responses.

### 3.2. Long-Term Maintenance of Immune Responses After Intranasal Vaccination

Given the durability of immune responses reported for adenoviral vector-based COVID-19 vaccines [25,26,27], we investigated whether this property is preserved following intranasal vaccination. Immunized mice were followed for 4 months after primary vaccination, with systemic immune responses longitudinally assessed in blood samples and responses in respiratory tract tissues evaluated at 4 months post-vaccination (Figure S1B).

#### 3.2.1. Antigen-Specific Antibody Responses in Serum and BALF

**Figure 3:**
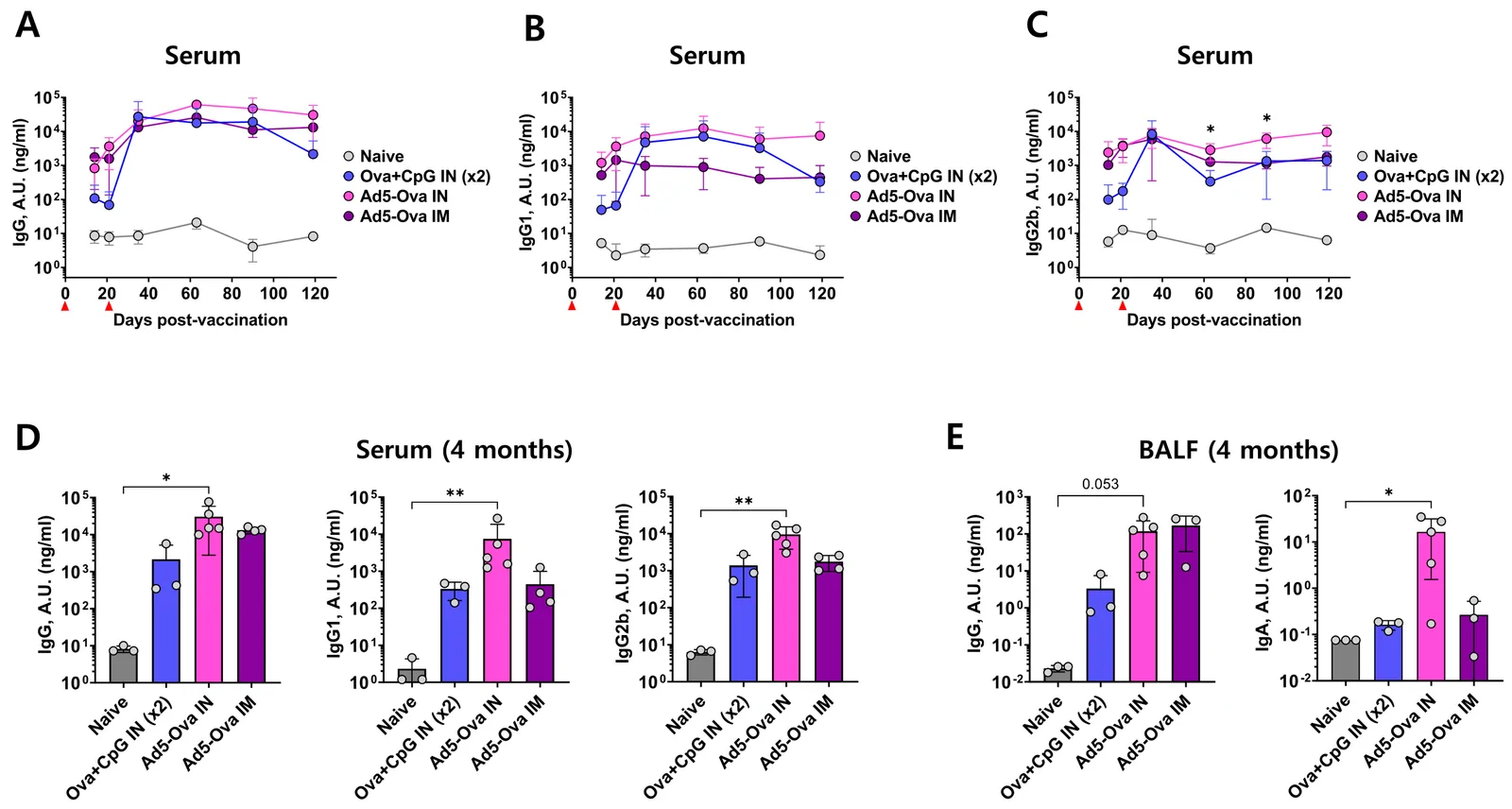
Long-term antibody responses after vaccination with CpG-adjuvanted or adenoviral vector vaccines. (**A**–**C**) Line graphs showing longitudinal Ova-specific IgG (**A**), IgG1 (**B**), and IgG2b (**C**) levels in serum. Red triangles indicate the day of vaccination. For statistical analysis, a mixed-effects model was used with Tukey’s multiple comparisons test. * *p* < 0.05, Ad5-Ova IN group vs. Ova+CpG IN (x2) group. (**D**,**E**) Ova-specific IgG subset levels in serum (**D**) or IgG and IgA levels in BALF (**E**) at 4 months post-vaccination. For statistical analysis, one-way ANOVA or the Kruskal–Wallis test was used, depending on normality. For multiple comparisons, Tukey’s test (one-way ANOVA) or Dunn’s test (Kruskal–Wallis test) was used. * *p* < 0.05; ** *p* < 0.01; Data are presented as the mean ± SD.

Ova-specific serum IgG levels, were longitudinally monitored for up to 4 months after primary vaccination (Figure 3A–C). Total Ova-specific IgG levels reached comparable peak levels across the groups, but during the final month, the Ad5-Ova groups maintained relatively stable responses, whereas the Ova+CpG IN (x2) group showed a gradual decline (Figure 3A). The Ad5-Ova IM group showed relatively lower IgG1 levels, while IgG1 in the Ova+CpG IN (x2) group also declined more rapidly than that in the Ad5-Ova IN group (Figure 3B). In contrast, IgG2b levels in the Ova+CpG IN (x2) group were maintained through 4 months after vaccination (Figure 3C). Overall, Ad5-Ova IN vaccination tended to maintain the highest levels of Ova-specific IgG responses compared with Ova+CpG IN (x2) and Ad5-Ova IM vaccination (Figure 3D). In BALF, both Ad5-Ova IN and Ad5-Ova IM vaccination induced higher Ova-specific IgG levels than Ova+CpG IN (x2) vaccination at 4 months post-vaccination. Notably, Ova-specific IgA levels were highest in the Ad5-Ova IN group (Figure 3E). These results demonstrate that intranasal adenoviral vector vaccination induces robust and sustained systemic and respiratory mucosal antibody responses for up to 4 months after a single vaccination.

#### 3.2.2. Antigen-Specific CD8 T Cell Responses in the mLNs, Lungs, and Blood

**Figure 4:**
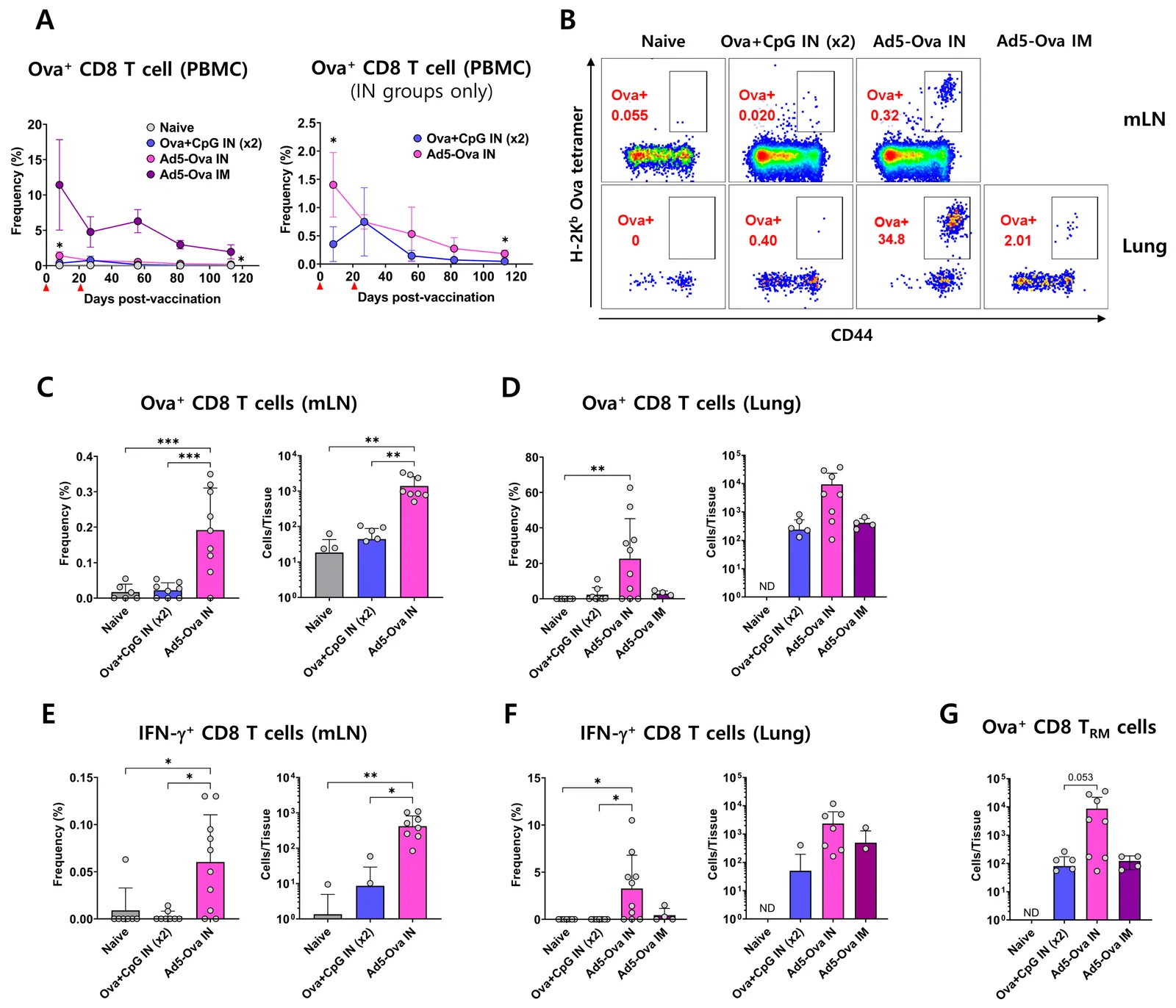
Long-term CD8 T cell responses after vaccination with CpG-adjuvanted or adenoviral vector vaccines. (**A**) Line graphs showing longitudinal Ova-specific CD8 T cell frequencies. Red triangles indicate the day of vaccination. For statistical analysis, a mixed-effects model was used with Tukey’s multiple comparisons test. * *p* < 0.05, Ad5-Ova IN group vs. Ova+CpG IN (x2) group. (**B**) Flow cytometry plots depicting Ova-specific CD8 T cells in the mLNs and lungs at 4 months post-vaccination. (**C**,**D**) Frequencies and numbers of Ova-specific CD8 T cells in the mLNs (**C**) and lungs (**D**). (**E**,**F**) Frequencies and numbers of IFN-γ-secreting CD8 T cells after ex vivo antigenic stimulation in the mLNs (**E**) and lungs (**F**). (**G**) Numbers of lung-resident memory CD8 T cells. For statistical analysis, one-way ANOVA or the Kruskal–Wallis test was used, depending on normality. For multiple comparisons, Tukey’s test (one-way ANOVA) or Dunn’s test (Kruskal–Wallis test) was used. * *p* < 0.05; ** *p* < 0.01; *** *p* < 0.001. Data are pooled from 2 independent experiments for Figure 4C–G. Data are presented as the mean ± SD.

We next assessed systemic and respiratory mucosal Ova-specific CD8 T cell responses up to 4 months after primary vaccination. IM administration of Ad5-Ova induced the highest levels of Ova-specific CD8 T cell responses in the blood throughout the observation period. Among the IN vaccination groups, Ad5-Ova vaccination induced rapid and sustained CD8 T cell responses in the blood (Figure 4A). In the respiratory mucosal tissues, Ova-specific CD8 T cells remained significantly higher in the mLNs and lungs of the Ad5-Ova IN group than in the Ova+CpG IN (x2) group at 4 months post-vaccination (Figure 4C,D). IFN-γ-secreting CD8 T cell responses showed a similar pattern to the Ova-specific CD8 T cell responses measured by class I MHC–peptide tetramer staining (Figure 4E,F). Because lung-resident memory T cells function as sentinels that detect pathogens entering via the respiratory tract and rapidly activate to protect against them, they are an important component of protective cellular immunity at the respiratory mucosa [28,29]. We therefore further analyzed lung-resident memory CD8 T cells (CD44^+^ Ova^+^ CD90.2^-^ CD69^+^ T cells) at 4 months post-vaccination. The Ad5-Ova IN group showed approximately 100-fold higher levels of lung-resident memory CD8 T cells than the other vaccination groups at this time point, although the difference did not reach statistical significance (Figure 4G). This trend may suggest a potential effect of intranasal Ad5-Ova vaccination on the induction of lung-resident memory CD8 T cells, although further studies are needed to confirm this finding.

Collectively, these results suggest that intranasal adenoviral vector vaccination induces sustained systemic and respiratory mucosal antigen-specific CD8 T cell responses and establishes a lung-resident CD8 T cell population that persists for at least 4 months after a single vaccination.

## 4. Discussion

In this study, a single intranasal dose of Ad5-Ova induced robust systemic and respiratory mucosal immune responses, including serum IgG, BALF IgA, and antigen-specific CD8 T cell responses in the lungs, which were maintained for up to 4 months after vaccination. Compared with the CpG-adjuvanted protein vaccine, these responses were generally stronger and more sustained following a single Ad5-Ova vaccination. These findings suggest that intranasal adenoviral vaccination has the potential to induce durable systemic and respiratory mucosal immunity following a single dose, addressing an important challenge in the development of effective intranasal vaccine strategies.

The superior and more durable immune responses induced by IN Ad5-Ova relative to CpG-adjuvanted Ova likely reflect fundamental differences between the two platforms in antigen kinetics and innate immune activation. Whereas CpG-adjuvanted protein is delivered as a bolus antigen that is rapidly cleared, adenoviral vectors transduce host cells and support de novo antigen synthesis over an extended period, providing a more sustained supply of antigen for uptake and presentation by antigen-presenting cells. This continuous antigen availability may help sustain Tfh and GC B cell responses, consistent with the enhanced and durable Tfh/GC responses observed in the mLNs following Ad5-Ova IN vaccination [30,31]. In addition, adenoviral vectors engage multiple innate sensing pathways—including recognition of the viral capsid, fiber protein, and cytosolic viral DNA via the cGAS-STING pathway—in contrast to CpG ODN, which signals predominantly through TLR9 alone [32,33]. In addition to the antigen availability, this broader activation of innate immunity may explain the stronger CD8 T cell priming observed after Ad5-Ova vaccination, as de novo intracellular antigen synthesis favors direct MHC class I loading, whereas protein-based vaccines depend more heavily on the comparatively less efficient cross-presentation of exogenous antigen. Consistent with this interpretation, CpG-adjuvanted Ova vaccination required a booster dose to achieve antibody and CD8 T cell responses comparable to those induced by a single dose of Ad5-Ova, suggesting that the GC and effector T cell responses initiated by CpG-adjuvanted protein contract more rapidly in the absence of sustained antigen and innate immune stimulation. These proposed mechanisms, however, were not directly tested in this study and warrant further investigation.

Our findings are consistent with several recent reports on single-dose intranasal adenoviral vaccination. Freitag et al. demonstrated that a single IN dose of Ad5 vectors expressing the SARS-CoV-2 spike protein or its receptor-binding domain induced robust BALF IgA and IgG responses, whereas intramuscular administration of the same vectors failed to induce IgA in either the serum or BALF, mirroring the route-dependence of mucosal IgA induction observed in our study [34]. Similarly, a simian adenovirus-based H1N1 vaccine induced IgA in the respiratory mucosa only after IN, and not IM, administration [35] further supporting the notion that mucosal IgA induction by adenoviral vectors depends critically on the route of delivery rather than the vector platform alone. In addition, a single IN dose of an adenovirus-based RSV vaccine targeting the G and M2 proteins maintained elevated BALF IgA and conferred protection against RSV challenge at 3 months post-immunization [36], supporting the potential for single-dose IN adenoviral vaccination to induce mucosal antibody responses that persist over a time frame comparable to that observed in our study. Notably, not all IN adenoviral vaccination studies report robust mucosal IgA induction. A recent study using a chimpanzee adenovirus (ChAd68)-vectored RSV prefusion F vaccine reported undetectable BALF IgA at 7 days after a single IN dose, despite conferring substantial protection against RSV challenge through apparently non-neutralizing antibody-mediated mechanisms [37]. This discrepancy with our findings may reflect differences in vector serotype (ChAd68 versus Ad5), antigen properties, or the earlier post-vaccination time point at which BALF was sampled, and highlights that the induction of mucosal IgA by adenoviral vectors is not a uniform property of the platform but may depend on vector- and antigen-specific factors that remain incompletely defined. Furthermore, whereas a study using IN Ad5 vectors expressing SARS-CoV-2 spike-derived antigens reported that systemic (splenic) antigen-specific T cell responses were short-lived, declining within 2 weeks despite durable mucosal IgA and airway memory T cell responses, our study observed antigen-specific CD8 T cell responses that remained elevated in the blood for up to 4 months after a single IN Ad5-Ova dose. This difference suggests that IN Ad5 vaccination can, under certain conditions, support more durable systemic T cell immunity than previously appreciated, although differences in antigen, readout tissue, and vaccination model preclude direct comparison. Collectively, these comparisons support further investigation of intranasal adenoviral vectors as a potential platform for inducing coordinated systemic and mucosal immunity, the magnitude and durability of these responses are likely shaped by vector- and antigen-specific factors that merit systematic investigation.

Several limitations of this study should be considered. First, Ova was used as a model antigen, and its use limits direct extrapolation of these findings to viral antigens, which may differ substantially in size, structure, glycosylation, and antigenic properties. Second, although we demonstrated antigen-specific antibody and CD8 T cell responses in respiratory tissues, the protective capacity of these responses was not directly evaluated. Third, the mechanisms underlying the potent and durable systemic and mucosal immune responses induced by IN adenoviral vaccination remain largely unclear and were not directly investigated in this study.

Future studies should address these limitations in several ways. Extending this vaccination strategy to clinically relevant antigens, such as influenza hemagglutinin or the SARS-CoV-2 spike protein, will be important for assessing the translational potential of single-dose IN adenoviral vaccination. Neutralizing antibody responses in serum and BALF should be evaluated, and protective efficacy should be directly tested using appropriate respiratory pathogen challenge models. Mechanistic studies—such as antigen persistence kinetics and profiling of innate immune activation between adenoviral vectors and CpG-adjuvanted protein—would help clarify the pathways underlying the differences in immunogenicity observed between platforms. Finally, because pre-existing immunity to human Ad5 is common in the human population, evaluating the impact of pre-existing anti-vector immunity on the immunogenicity of IN Ad5-based vaccines will be an important consideration for clinical translation.

## 5. Conclusions

A single intranasal dose of Ad5-Ova induced robust and sustained systemic and respiratory mucosal immune responses in mice, including antigen-specific antibody and T cell responses that remained detectable for up to 4 months, as well as Ova-specific CD8 T cells with a lung-resident memory phenotype. In comparison, CpG-adjuvanted protein vaccination induced weaker responses after a single dose and required a booster to achieve comparable short-term immunogenicity. Together, these findings indicate that a single intranasal dose of Ad5-Ova can induce sustained systemic and respiratory mucosal immune responses, including lung-resident CD8 T cells, and highlight the potential of adenoviral vector vaccination as an approach for single-dose mucosal immunization. Further studies using clinically relevant respiratory pathogen antigens and appropriate challenge models are warranted to determine whether these immune responses translate into protective efficacy.

## Data Availability

The original contributions presented in this study are included in the article. Further inquiries can be directed to the corresponding author(s).

## Supplementary Materials

The following supporting information can be downloaded at: https://www.mdpi.com/article/10.3390/vaccines14090793/s1, Figure S1: Schematic representation of the immunization and sample collection schedule; Figure S2: Cellular immune responses in mLNs at 2 weeks post-vaccination; Figure S3: Cellular immune responses in the lungs at 2 weeks post-vaccination.

## Abbreviations

The following abbreviations are used in this manuscript:

COVID-19	Coronavirus disease of 2019
Ig	Immunoglobulin
sIgA	Secretory IgA
CpG ODN	CpG oligodeoxynucleotides
mLN	Mediastinal lymph node
BALF	Bronchoalveolar lavage fluid
IN	Intranasal
IM	Intramuscular
Ad5	Adenovirus serotype 5-vector
Ova	Ovalbumin
HRP	Horseradish peroxidase
FACS	Fluorescent activated cell sorting
MHC	Major histocompatibility complex
T_fh_	Follicular helper T cell
GC	Germinal center
T_RM_	Tissue-resident memory T cell
