# Supplementary material for: Intranasal Adenoviral Vector Vaccination Induces Durable Antibody and T Cell Responses in the Respiratory Tract
Source: Vaccines (Basel). 2026 Sep 9;14(9):793. doi: 10.3390/vaccines14090793 (PMC13611307; doi:10.3390/vaccines14090793)
Supplement: Supplementary file 1 [file vaccines-14-00793-s001.zip › vaccines-4530000-supplementary.pdf]

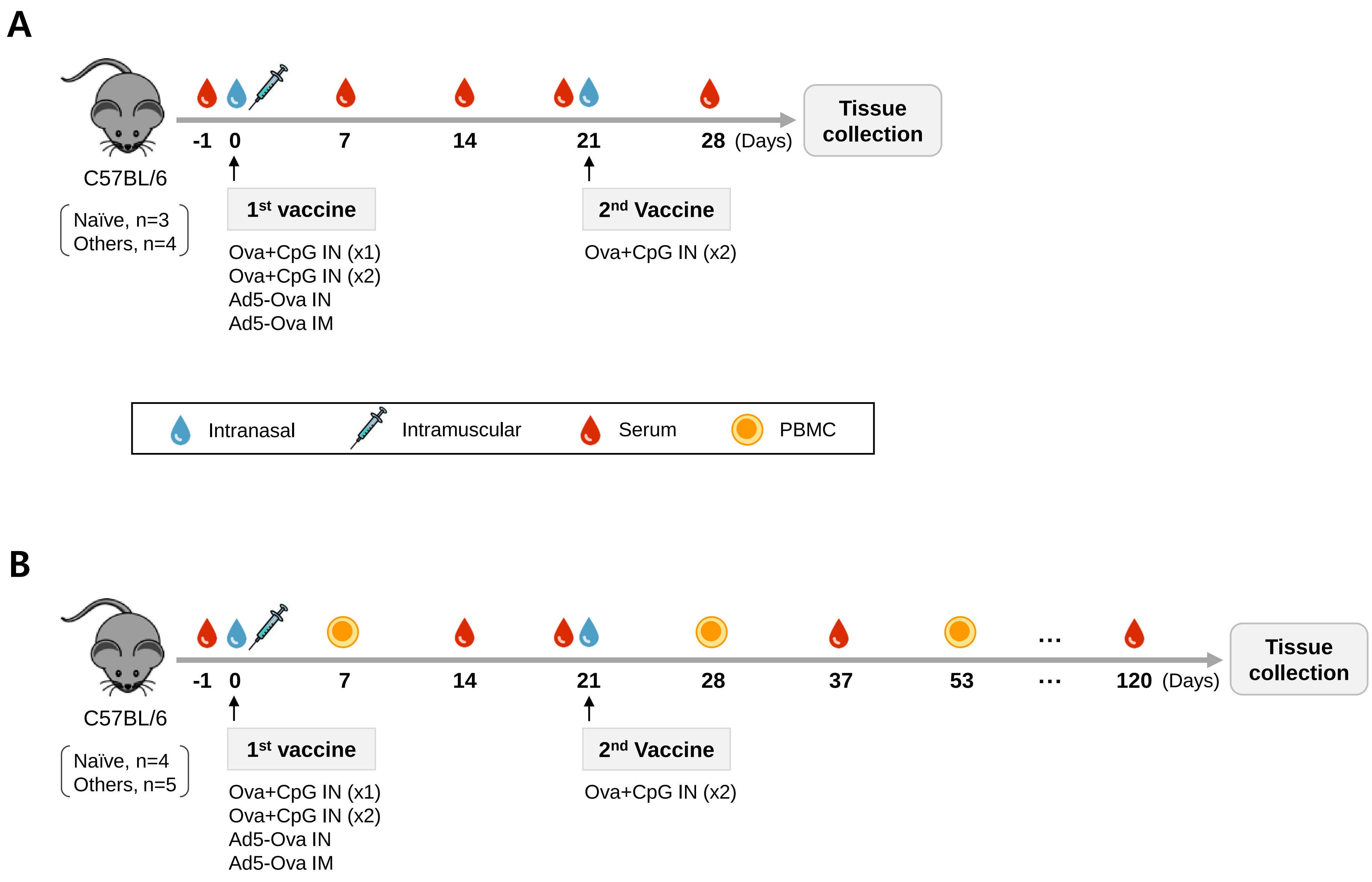

Supplementary Figure S1. Schematic representation of the immunization and sample collection schedule. (**A**) Short-term immunogenicity study (**B**) Long-term immunogenicity study. Each experimental set was inde pendently repeated twice.

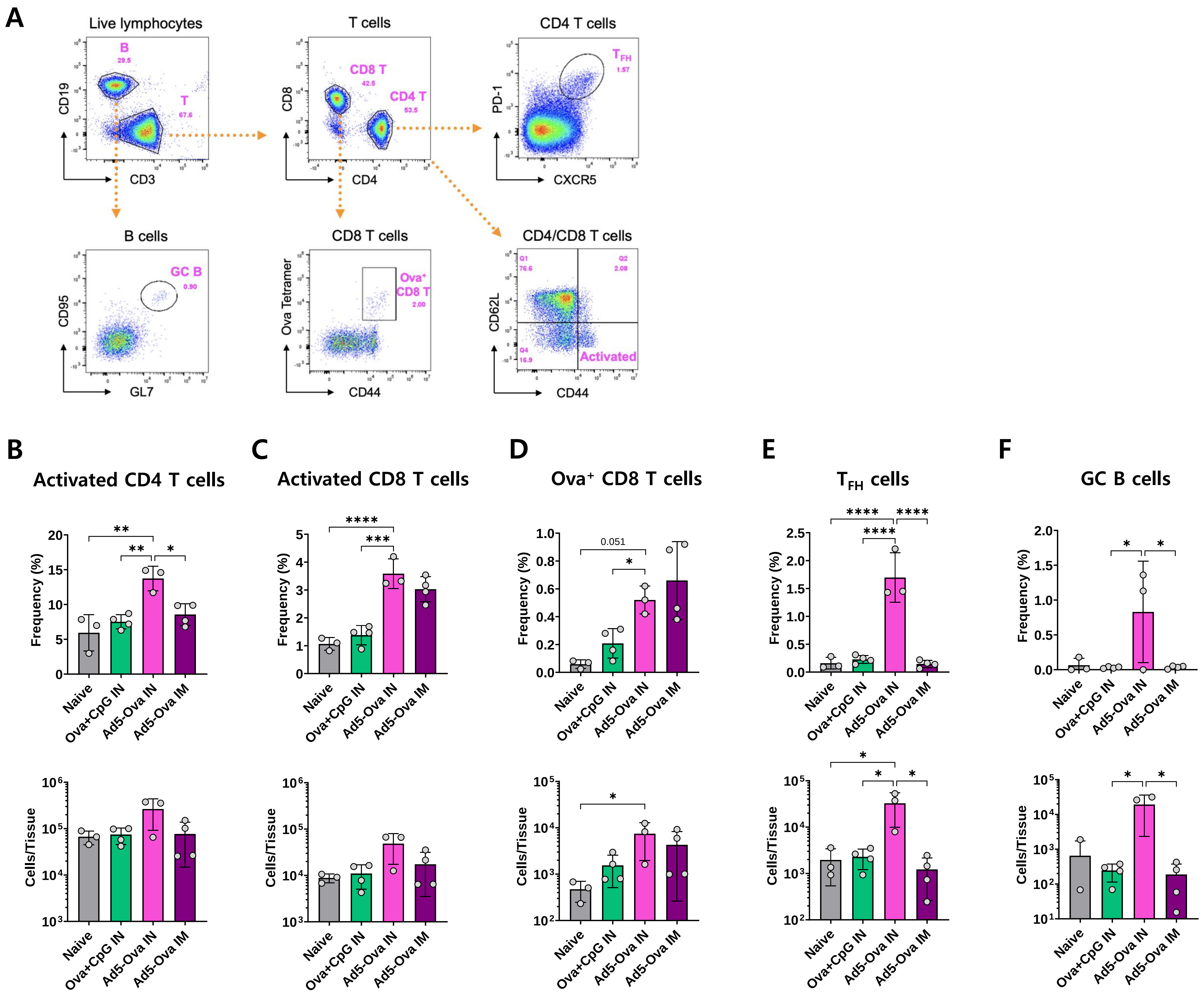

Supplementary Figure S2. Cellular immune responses in mLNs at 2 weeks post-vaccination. (**A**) Representat ive flow cytometry gating strategy for lymphocytes, (**B-F**) Frequencies and cell counts of activated CD4 T cel ls (**B**), CD8 T cells (**C**), Ova-specific CD8 T cells (**D**), follicular helper T cells (**E**), and germinal center B cells (**F**) in the mLNs at 2 weeks post-vaccination. For statistical analysis, one-way ANOVA, Welch's ANOVA, or the Kr uskal-Wallis test was used, depending on normality and equality of variances. For multiple comparisons, Tu key's test (one-way ANOVA), Dunnett's T3 test (Welch's ANOVA), or Dunn's test (Kruskal-Wallis test) was use d. *P<0.05; **P<0.01; ***P<0.001; ****P<0.0001. Data are presented as the mean ± SD.

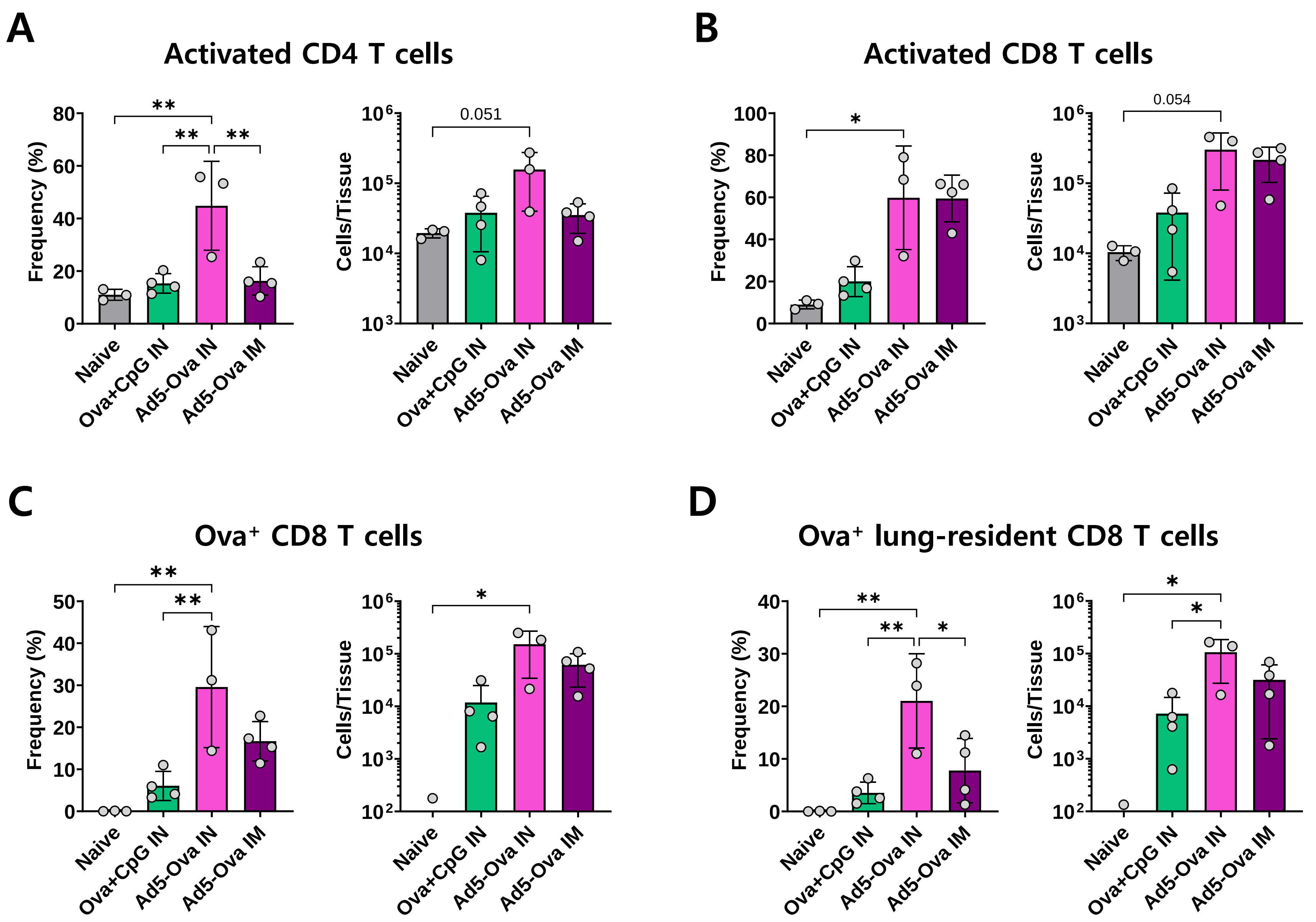

Supplementary Figure S3. Cellular immune responses in lungs at 2 weeks post-vaccination. (**A-D**) Frequencies and numbers of activated CD4 T cells (**A**), CD8 T cells (**B**), Ova-specific CD8 T cells (**C**) , and lung-resident (CD90.2^{-}) Ova-specific CD8 T cells (**D**) in the lungs at 2 weeks post-vaccination . For statistical analysis, one-way ANOVA or the Kruskal-Wallis test was used, depending on norm ality. For multiple comparisons, Tukey's test (one-way ANOVA) or Dunn's test (Kruskal-Wallis test) was used. *P<0.05; **P<0.01. Data are presented as the mean ± SD.
